# The JAK-STAT Transcriptional Regulator, STAT-5, Activates the ATM DNA Damage Pathway to Induce HPV 31 Genome Amplification upon Epithelial Differentiation

**DOI:** 10.1371/journal.ppat.1003295

**Published:** 2013-04-04

**Authors:** Shiyuan Hong, Laimonis A. Laimins

**Affiliations:** Department of Microbiology-Immunology, Northwestern University, Feinberg School of Medicine, Chicago, Illinois, United States of America; National Institute of Allergy and Infectious Diseases, National Institutes of Health, United States of America

## Abstract

High-risk human papillomavirus (HPV) must evade innate immune surveillance to establish persistent infections and to amplify viral genomes upon differentiation. Members of the JAK-STAT family are important regulators of the innate immune response and HPV proteins downregulate expression of STAT-1 to allow for stable maintenance of viral episomes. STAT-5 is another member of this pathway that modulates the inflammatory response and plays an important role in controlling cell cycle progression in response to cytokines and growth factors. Our studies show that HPV E7 activates STAT-5 phosphorylation without altering total protein levels. Inhibition of STAT-5 phosphorylation by the drug pimozide abolishes viral genome amplification and late gene expression in differentiating keratinocytes. In contrast, treatment of undifferentiated cells that stably maintain episomes has no effect on viral replication. Knockdown studies show that the STAT-5β isoform is mainly responsible for this activity and that this is mediated through the ATM DNA damage response. A downstream target of STAT-5, the peroxisome proliferator-activated receptor γ (PPARγ) contributes to the effects on members of the ATM pathway. Overall, these findings identify an important new regulatory mechanism by which the innate immune regulator, STAT-5, promotes HPV viral replication through activation of the ATM DNA damage response.

## Author Summary

Over 120 types of human papillomavirus (HPV) have been identified, and approximately one-third of these infect epithelial cells of the genital mucosa. A subset of HPV types are the causative agents of cervical and other anogenital cancers. The infectious life cycle of HPV is dependent on differentiation of the host epithelial cell, with viral genome amplification and virion production restricted to differentiated suprabasal cells. While normal keratinocytes exit the cell cycle upon differentiation, HPV-positive suprabasal cells are able to re-enter S-phase to mediate productive replication. HPV induces an ATM-dependent DNA damage response in differentiating cells that is essential for viral genome amplification. Our studies describe an important mechanism by which human papillomaviruses activate a member of the JAK/STAT innate immune signaling pathway to induce the ATM DNA damage pathway. This is necessary for differentiation-dependent productive viral replication. HPVs must suppress the transcription of one member of the JAK/STAT pathway, STAT-1, while at the same time activating STAT-5 to regulate genome amplification in suprabasal cells. The E7 protein activates STAT-5 leading to induction of ATM phosphorylation through the PPARγ pathway. Our study identifies important links between innate immune signaling, the ATM DNA damage pathway and productive HPV replication that may lead to the characterization of new targets for the development of therapeutics to treat HPV-induced infections.

## Introduction

Human papillomaviruses (HPVs) are the causative agents of cervical and other anogenital cancers [1]. Over 120 types of HPVs have been identified and approximately one third of these types infect the squamous epithelia of the genital tract. High-risk genital HPVs including HPV16, 18, 31, and 35 are sexually transmitted. HPVs infect cells in the basal layer of stratified epithelia and virion production is dependent upon epithelial differentiation [2]. To establish persistent infection in basal cells, HPVs must escape host innate immune surveillance as well as the adaptive immune response through mechanisms that are not yet understood. High-risk HPV genomes encode approximately six early genes and two late genes. The E6 and E7 genes encode oncoproteins that play important roles in regulation of the productive life cycle as well as in the development of anogenital cancers [2], [3]. E6 has many activities including the recruitment of the cellular E3 ubiquitin ligase E6-associated protein (E6AP) into a trimeric complex with p53 that results in its degradation [4]–[6]. E7 protein binds to several cellular factors such as the retinoblastoma protein (Rb) leading to the constitutive activation of E2F family members [7], [8]. Both E6 and E7 have been implicated as important regulators of immune evasion [9].

The HPV life cycle is closely associated with epithelial differentiation. Following initial infection, HPV viral genomes are maintained as low-copy episomes in undifferentiated basal cells. As HPV-infected cells differentiate, the late viral promoter is activated. This results in enhanced expression of viral replication proteins, E1 and E2, along with L1 and L2 capsid proteins[10]–[13]. The amplification of viral genomes is similarly induced upon differentiation in suprabasal epithelial cells [14] resulting in virion production and release. The amplification of HPV genomes in differentiating cells is dependent on both viral and host factors such as polymerases and transcription factors [2] as well as with members of the ataxia-telangiectasia mutated (ATM) kinase pathway [15]. Activation of the ATM DNA damage pathway has been shown to be necessary for HPV genome amplification in differentiating cells but has no effect on the stable maintenance of episomes in undifferentiated cells [15]. In normal cells, double strand breaks are recognized by the trimeric MRN complex, which leads to the activation of the ATM kinase by autophosphorylation and its recruitment into distinct nuclear foci [16]. ATM kinases activate a series of downstream effectors including the kinase CHK2, histone γ-H2AX along with BRCA1 [17]. These factors are activated in HPV infections but how they regulate HPV genome amplification and what signals are responsible for their activation remains unclear.

To establish persistent infection, HPVs must evade surveillance by both innate and adaptive immune responses. One of the primary pathways regulating the innate immune response is the JAK/STAT pathway [18]. The Janus kinase-signal transducer and activator of transcription (JAK/STAT) pathway is activated by external growth signals mediated through cytokines, growth factors and interferons leading to translocation of STAT proteins to the nucleus. This leads to the increased expression of hundreds of downstream genes [19], [20]. The STAT family of proteins includes STAT-1, -2, -3, -4, -5, and -6. Recent studies have shown that HPV gene products suppress transcription of STAT-1, but not STAT-2 or STAT-3, and that this is necessary for stable maintenance of viral episomes and genome amplification [9]. STAT-5 consists of two distinct isoforms, STAT-5α and STAT-5β that form homo or heterodimers. Knockout mice lacking both STAT-5α and STAT-5β exhibit a perinatal lethal phenotype [21] and have severely impaired lymphoid development and differentiation [22]. We investigated if the levels of STAT-5, like STAT-1, were reduced in HPV positive cells and whether these innate immune regulators play any role in HPV pathogenesis. Our studies reveal that STAT-5 is activated in HPV positive cells and that it is necessary for HPV genome amplification. STAT-5 regulates genome amplification through activation of the ATM DNA damage response that is mediated, in part, through the peroxisome proliferator-activated receptor γ (PPARγ).

## Results

### STAT-5 is constitutively activated in HPV-infected keratinocytes

**Figure 1:**
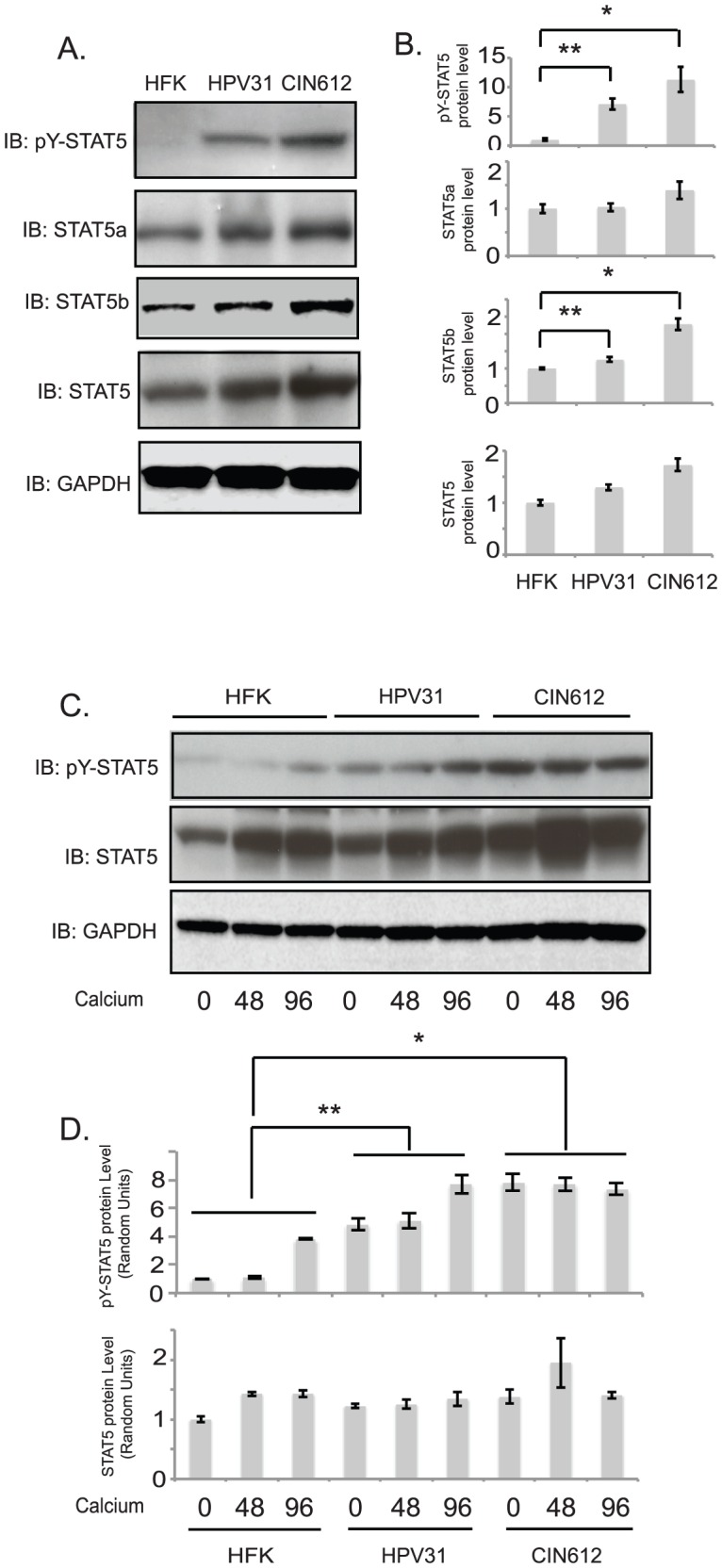
HPV31 increases the levels of STAT-5 phosphorylation. A) Western blot analysis of STAT-5, p-STAT-5, and GAPDH levels in HFK, HPV31, and HPV31-positive CIN612 cells grown in monolayer cultures. B) Bar graphs show the relative expression level of target proteins, normalized to GAPDH, from panel A western analysis. The statistical analysis was assayed by 2-tail t-test. Data = mean +/−standard error. * indicates p-value<0.01; ** indicates p-value<0.05. The band intensities were determined by ImageJ64 software. C) Western blot analysis for STAT-5, p-STAT-5, and GAPDH levels in HFK, HPV31 and HPV31-positive CIN612 cells differentiated in high calcium media for indicated times. D) Bar graphs show the relative expression level of target proteins, normalized to GAPDH, from panel C western analysis. The statistical analysis was assayed by 2-tail t-test. Data = mean +/−standard error. * indicates p-value<0.01; ** indicates p-value<0.05. All results are representative of observations from 3 independent experiments.

Human papillomaviruses must escape surveillance by the innate immune system to establish persistent infections. Previous studies demonstrated that high-risk HPVs suppress expression of the interferon pathway activator, STAT-1 but not STAT2 or STAT3 and that this repression is necessary for stable maintenance of viral genomes as well as differentiation-dependent genome amplification [9]. We were interested in investigating if HPV proteins similarly suppressed other members of the JAK-STAT pathway. STAT-5 proteins share significant homology with STAT-1 but regulate expression of a different set of effector genes. In addition, STAT5 is activated by cytokines and growth factors along with interferons. To determine if HPV proteins altered the total levels of STAT-5, extracts of HPV positive keratinocytes and normal human keratinocytes were examined by Western blot analysis. In contrast to the suppression seen with STAT-1, we found no significant differences in STAT-5 levels between normal keratinocytes and stably transfected HPV31-positive keratinocytes or cells derived from an HPV31-positive biopsy (CIN 612: Figure 1A). STAT-5 is activated by phosphorylation following exposure to cytokines or growth factors and it was important to investigate if HPV proteins altered the levels of phosphorylated STAT-5. Our studies demonstrated significantly increased levels of phosphorylated forms of STAT-5 in HPV31-positive cells as compared to normal cells. Interestingly, this activation was observed in the absence of any added cytokines or growth factors. This suggested that constitutive activation of STAT-5 by HPV proteins could play a role in the HPV life cycle.

To determine if STAT-5 levels changed during the differentiation-dependent life cycle of HPV, we examined the levels of STAT-5 in HPV31-positive and -negative keratinocytes grown in high-calcium media to induce differentiation. As shown in Figure 1B, the levels of STAT-5 increase upon differentiation of both HFKs and HPV31-positive cells, with slightly higher levels in HPV positive cells (Figure 1B). Importantly, the active, phosphorylated forms of STAT-5 are present at increased levels in differentiating HPV31-positive cells relative to HFKs. The expression of keratin 10 (K-10), a member of intermediate filaments, and involucrin were used as markers of differentiation (not shown).

### STAT5 inhibition by pimozide blocks differentiation-dependent HPV31 genome amplification and late gene expression

**Figure 2:**
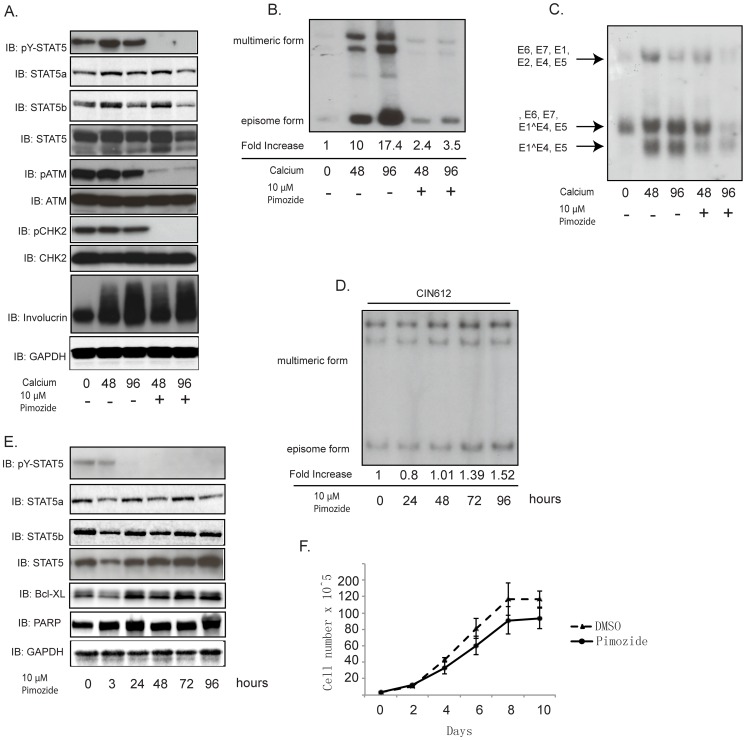
Suppression of STAT-5 phosphorylation by pimozide blocks HPV genome amplification upon keratinocyte differentiation. A) Western blot analysis of p-STAT-5, STAT-5α, STAT-5β, STAT-5, p-ATM, ATM, p-CHK2, CHK2 and GAPDH proteins in differentiated CIN612 cells in the presence or absence of pimozide (10 µM) in high calcium media for indicated times. B) Southern blot analysis for HPV31 episomes in CIN612 cells untreated or treated with pimozide (10 µM) following differentiation in high calcium media for indicated times (hrs). C) Northern blot analysis for HPV31 early and late gene expression in CIN612 cells in the presence or absence of pimozide (10 µM) following differentiation in high calcium media for indicated times (hrs). D) Southern blot analysis for HPV31 episomes in monolayer cultures of CIN612 cells untreated or treated with pimozide (10 µM) for indicated times (hrs). E) Western blot analysis of p-STAT-5, STAT-5α, STAT-5β, STAT-5, Bcl-XL, and PARP proteins in monolayer CIN612 cells treated with pimozide (10 µM) for indicated times. F) Cell proliferation assay of monolayer CIN612 cells with or without pimozide (10 µM) for indicated times. P<0.05. All results are representative of observations from 2 or more independent experiments.

Since our studies indicated that the levels of phospho-STAT-5 are significantly increased in HPV positive cells, it was important to determine if activated STAT-5 played any role in the differentiation-dependent viral life cycle. Pimozide is an inhibitor of STAT-5 activation [23] and we investigated what effect treatment with this drug had on differentiation-dependent HPV31 genome amplification and late gene expression. HPV31-positive CIN 612 cells were treated with pimozide for 12 hours and then transferred to high-calcium media in the continued presence of pimozide for an additional 48 and 96 hours. As seen in Figure 2A, phosphorylation of STAT-5 in HPV31-positive cells was suppressed by pimozide upon differentiation, however, treatment had no effect on total STAT-5 protein levels. STAT-5 consists of two comparably expressed isoforms and the levels of both forms were unaltered by pimozide treatment (Figure 2A). In addition, pimozide had no effect on involucrin expression upon differentiation (Figure 2A). We next investigated if pimozide treatment had any effect on HPV differentiation-dependent late functions. Amplification of HPV 31 genomes begins at approximately 48 hours after the addition of high calcium media and plateaus by 96 hours. Total DNA was isolated from treated and untreated CIN 612 cells after 48 and 96 hours of differentiation and examined for viral genome amplification by Southern blot analysis. As seen in Figure 2B, treatment with pimozide significantly reduced amplification of viral genomes upon keratinocyte differentiation. Total RNA was also isolated from pimozide-treated HPV31-positive keratinocytes and examined for viral late gene expression by Northern blot analysis. In untreated HPV positive cells, high levels of the major late viral transcripts encoding E1∧E4, and E5, were observed at 48 hours of differentiation and pimozide treatment was found to block viral late gene expression (Figure 2C). This indicates that STAT-5 plays an important role for both HPV genome amplification and for late gene expression (Figure 2B&2C). To address whether pimozide interferes with HPV genome maintenance in undifferentiated cells, total DNA from treated and untreated monolayer cells was isolated at different times and screened by Southern blot analysis. As shown in Figure 2D, pimozide has a minimal effect on HPV genome maintenance in undifferentiated cells.

To exclude the possibility that the loss of genome amplification or late gene expression upon pimozide treatment is due to alterations in cell growth or induction of apoptosis, we grew cells in the presence of pimozide and screened for apoptotic or anti-apoptotic markers by Western blot analysis (Figure 2E & 2F). Figure 2E shows that pimozide treatment specifically suppresses the phosphorylation of STAT-5 within 24 hours but has no effect on the levels of total STAT-5α or STAT-5β. In addition, we did not observe any significant changes in levels of full-length or cleaved PARP-1, an apoptotic marker, or Bcl-XL, an anti-apoptotic marker. Similarly, the growth rates of cells treated with pimozide are comparable to non-treated cells (Figure 2F). These results indicate that the effect of pimozide in blocking HPV31 genome amplification is due to inhibition of STAT-5 phosphorylation.

### STAT-5 knockdown by shRNA studies blocks HPV31 genome amplification

**Figure 3:**
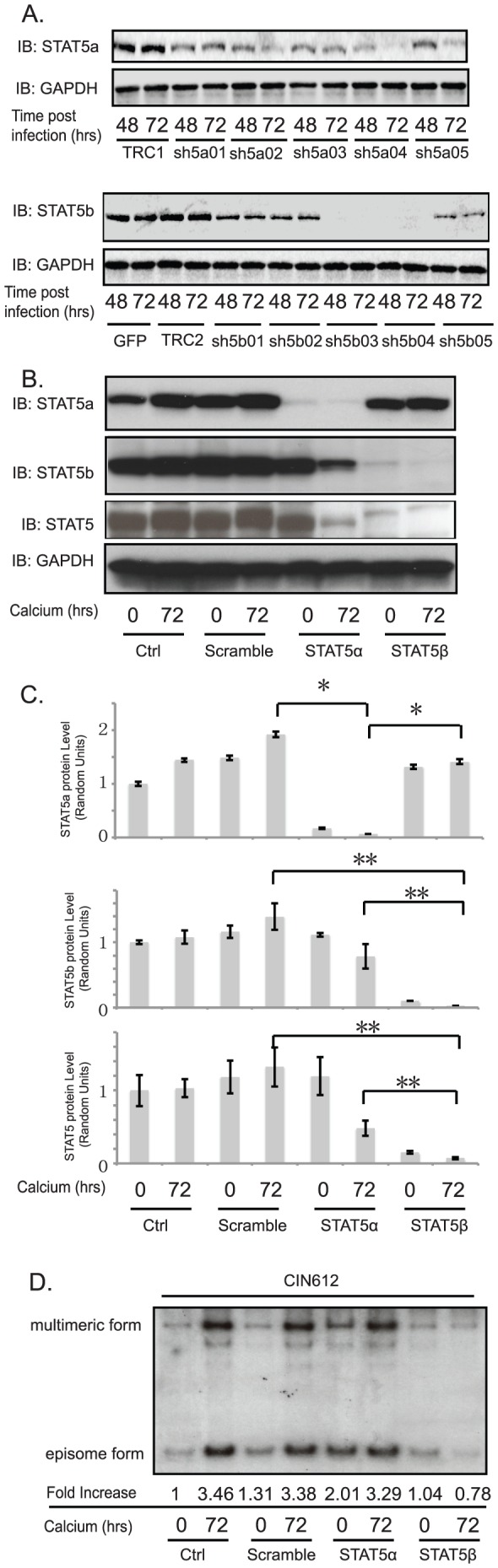
STAT-5 knockdown results in a loss of differentiation-dependent HPV genome amplification. HPV31-positive CIN612 cells were infected with lentiviruses expressing scramble shRNA, STAT-5α shRNA, or STAT-5β shRNA and incubated for 48 or 72 hours postinfection, followed by an additional 72 hours of differentiation in high calcium media. A) Western blot analysis of STAT-5α, STAT-5β, and STAT-5 proteins in monolayer CIN612 cells infected with multiple shRNA lentiviruses targeting STAT-5α or STAT-5β for indicated times. TRC1 and TRC2 refer to the scrambled vector controls for STAT-5α and STAT-5β respectively. B) Western blot analysis of STAT-5α, STAT-5β, and STAT-5 proteins following differentiation of CIN612 cells that had been infected with combined shRNA lentiviruses targeting STAT-5α or STAT-5β for indicated times. C) Bar graphs show the relative expression level of target proteins, normalized to GAPDH, from panel B western analysis. The statistical analysis was assayed by 2-tail t-test. Data = mean+/−standard error. * indicates p-value<0.01; ** indicates p-value<0.05. D). Southern blot analysis of HPV31 genomes in CIN612 cells following infection with shRNA lentiviruses and differentiation in high calcium media for indicated times (hrs). All results are representative of observations from 2 or more independent experiments.

It was important to confirm that the effects on HPV amplification observed with pimozide were specific for STAT-5 through knockdown studies using lentiviruses expressing shRNAs. As mentioned, STAT-5 has two isoforms and we knocked down each isoform separately. This allowed us to also determine whether the effects were specific to one of these isoforms. We first transfected the lentiviral vectors specific for either STAT-5α or STAT-5β into 293T cells to generate the corresponding recombinant viruses. Monolayer cultures of HPV31-positive CIN 612 cells were then infected with a series of these recombinant lentiviruses expressing shRNAs targeting STAT-5α or STAT-5β individually. At 48 hours or 72 hours after transduction, cell lysates were harvested and assayed for STAT-5 protein levels by Western blot analysis. Our data showed that two of the five STAT-5α-specific shRNAs, sh5a02 and sh5a04, significantly reduced the levels of STAT-5α in monolayer CIN 612 cells (Figure 3A). Similarly, two of the five STAT-5β-specific shRNAs, sh5b03 and sh5b04, were found to decrease the levels of STAT-5β. For the subsequent experiments, we pooled two of the isoform specific shRNAs together to knock down STAT-5α or STAT-5β individually. HPV31-positive CIN 612 cells were infected with shRNA-expressing lentiviruses, followed by differentiation in high calcium media for 72 hours and lysates were harvested for Western or Southern blot analysis. In control HPV31-positive cells or cells infected with scrambled shRNA-expressing lentiviruses, the levels of STAT-5α and STAT-5β are unchanged upon differentiation (Figure 3B). In contrast, in cells transduced with shRNAs targeting STAT-5β, the levels of STAT-5β were reduced with no effect on STAT-5α. Cells infected with lentiviruses expressing shRNAs targeting STAT-5α reduced STAT-5α levels but also moderately affected levels of STAT-5β. Cells infected with lentiviruses expressing shRNAs targeting STAT-5β reduced the total STAT-5 levels as we believed that STAT-5β is the major isoform. Southern blot analysis of these cells indicated that the loss of STAT-5β greatly impairs HPV31 viral amplification upon differentiation while STAT-5α knockdowns showed increased genomes in undifferentiated cells and modestly impaired amplification (Figure 3C).

### STAT-5 knockdown by shRNA inhibits ATM DNA damage activation

Activation of the ATM DNA damage pathway has been shown to be necessary for HPV genome amplification upon differentiation [15]. In order to understand what role STAT-5 plays in regulating HPV genome amplification, we investigated whether there is any change in activation of the ATM DNA damage pathway in cells in which phopsho-STAT-5 levels were reduced following treatment with pimozide. The levels of phosphorylated ATM and CHK2 are present at high levels in both undifferentiated and differentiated HPV positive keratinocytes in contrast to low levels in HFKs. The levels of total ATM in HPV positive cells were not changed by treatment with pimozide, however the levels of phosphorylated ATM were significantly reduced. Importantly, similar effects were seen with total and phosphorylated CHK2 (Figure 2A), which we have previously shown to be important for HPV genome amplification [15]. This indicates that phosphorylation of STAT-5 is important for activation of the ATM DNA damage pathway including p-CHK2 in HPV positive cells.

**Figure 4:**
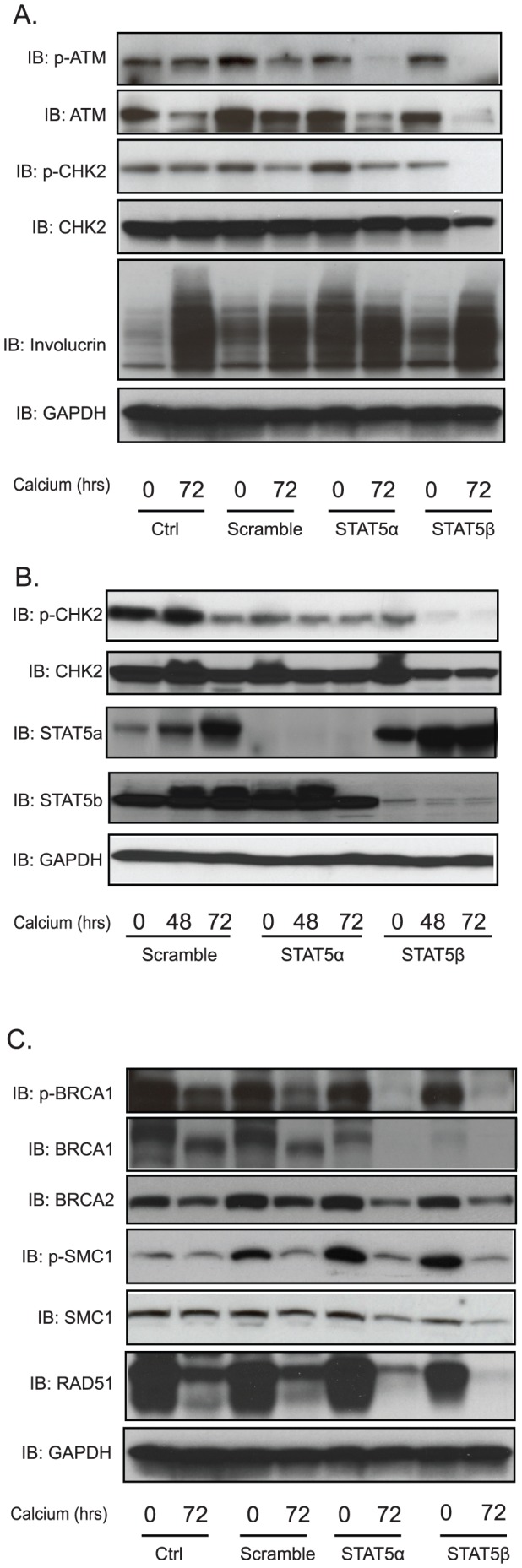
Knockdown of STAT-5 suppresses ATM DNA damage responses. HPV31-positive CIN612 cells were transduced as described in legend to Figure 3. A) Western blot analysis of p-ATM, ATM, p-CHK2, CHK2, involucrin and GAPDH protein levels in uninfected and shRNA lentivirus infected CIN612 cells upon differentiation in high-calcium media for indicated times. B) Western blot analysis of p-CHK2, CHK2, STAT-5α, STAT-5β, and GAPDH protein levels in shRNA control and shRNA lentivirus infected CIN612 cells upon differentiation in high-calcium media for indicated times. C) Western blot analysis of BRCA-1. BRCA-2, SMC-1, p-SMC-1, RAD51 and GAPDH protein at total or phosphorylation levels in uninfected and shRNA lentivirus infected CIN612 cells upon differentiation for indicated times. The quantification of the band intensities is shown as bar graph figures in Figure S1. All results are representative of observations from 3 independent experiments.

We next wanted to investigate the effects on activation of the ATM DNA damage pathway in STAT-5 knockdown cells through Western blot analysis. As shown in Figure 4A, the levels of total and phosphorylated forms of ATM are unchanged upon differentiation of either non-transduced cells or cells infected with scramble shRNA control lentiviruses. In contrast, the total levels of ATM are decreased in both STAT-5α or STAT-5β knockdown cells after infection and 72 hours of differentiation in high-calcium media. Similarly the phosphorylated forms of ATM are also reduced after 72 hours, however, significant levels of p-ATM were retained in the STAT-5α knockdowns while none was detected in the STAT-5β knockdowns. Previous studies using inhibitors to CHK2 indicated it was a key regulator of genome amplification [15] and we next investigated if it was altered by knockdown of STAT-5 isoforms. The levels of total CHK2 were only modestly reduced by STAT-5α or STAT-5β knockdown. In contrast, the phosphorylated form of CHK2 was significantly reduced in the STAT-5β knockdowns upon differentiation while this was not seen in the STAT-5α knockdowns. This indicates that STAT-5β is important for activation of CHK2. While the levels of p-ATM are reduced at the 72-hour point in the STAT-5α knockdowns, our data indicates that sufficient levels are present to maintain high levels of phosphorylated CHK2. It is also possible that another kinase, such as ATR, contributes to phosphorylation of CHK2 in the STAT-5α knockdown. As shown in Figure 4B, we confirmed that knockdown of STAT-5β resulted in loss of CHK2 phosphorylation at 48 hours as well as 72 hours of differentiation with minimal reductions seen in STAT-5α knockdowns. We believe that CHK2 may be the primary regulator of HPV genome amplification and that it is regulated in large part through STAT-5β.

Additional downstream substrates of the DNA damage response such as BRCA2 and SMC-1 were not changed by STAT-5 knockdown (Figure 4B). In contrast, the levels of BRCA-1 and phospho-BRCA-1 were reduced by STAT-5 knockdown upon differentiation. Another member of DNA damage response RAD51 was also suppressed by STAT-5 knockdown in the differentiating cells. Importantly, knockdown of STAT-5β or STAT-5α did not change the expression of involucrin, a marker of keratinocyte differentiation (Figure 4A). This analysis confirms the observations using inhibitors and further suggests that unphosphorylated STAT-5 isoforms may influence the levels of total protein levels as well as activation status of the ATM pathway members.

### PPARγ is downstream of STAT-5 signaling and regulates DNA damage responses

**Figure 5:**
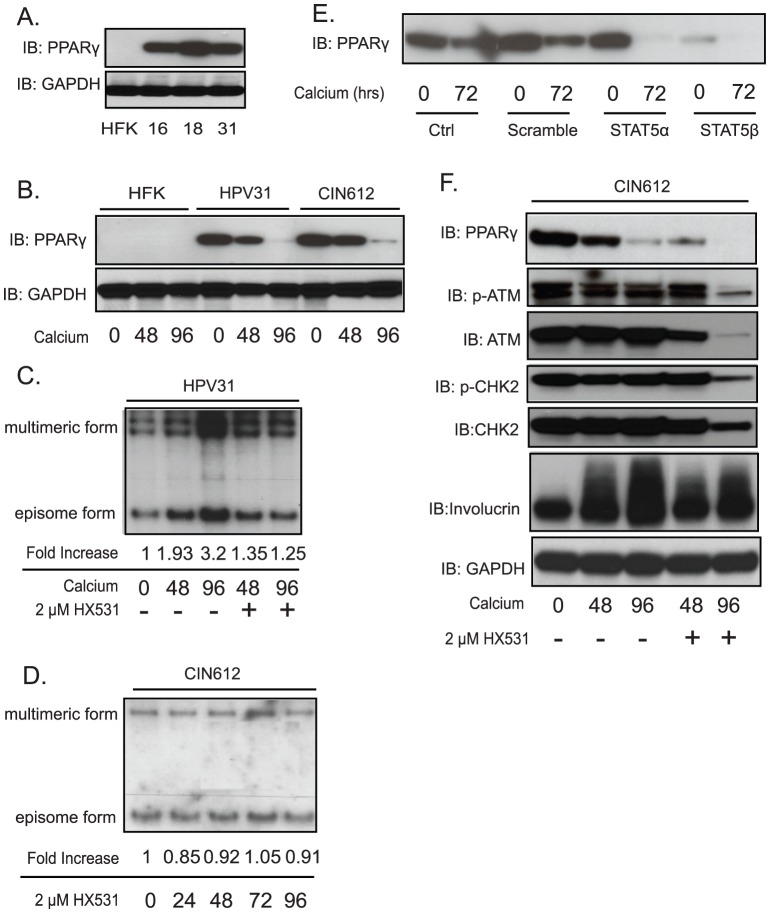
STAT-5-dependent ATM DNA damage signaling may be mediated by PPARγ. A) Western blot analysis of PPARγ and GAPDH protein levels in HFKs and HPV-31 positive keratinocytes. B) Western blot analysis for PPARγ and GAPDH levels in HFK, HPV31 and HPV31-positive CIN612 cells differentiated in calcium media for indicated times. C) Southern blot analysis for HPV31 episomes in HPV31 cells or the cells exposed to HX531 (2 µM) following differentiation in high calcium media for indicated times (hrs). D) Southern blot analysis for HPV31 episomes in CIN612 cells or the cells exposed to HX531 (2 µM) for indicated times (hrs). E) Western blot analysis of PPARγ protein levels in differentiating CIN612 cells infected with multiple shRNA targeting STAT-5α or STAT-5β for indicated times. F) Western blot analysis of PPARγ, ATM, CHK2, ATR, and CHK1 proteins at total or phosphorylation levels in differentiated CIN612 cells treated with or without HX531 (2 µM) in high calcium media for indicated times. The quantification of the data in bar figures is shown in Figure S2. All results are representative of observations from 3 independent experiments.

We next investigated the mechanism by which STAT-5 regulates DNA damage responses in HPV-positive keratinocytes. A recent study suggested that the nuclear peroxisome proliferator-activated receptor γ (PPARγ) could activate CHK2 in the development of follicular thyroid cancer [24]. In addition, PPARγ transcription has been shown to be regulated by STAT-5 in adipogenesis [25]. We therefore investigated if the expression of PPARγ was altered by STAT-5 in HPV31-positive CIN612 cells. We first examined the level of PPARγ in HPV-positive cells and found that PPARγ is expressed at high levels in HPV16, 18, and 31 positive cells as compared to HFKs (Figure 5A). Upon differentiation, the level of PPARγ decreases in HPV31-positive cells, but is still expressed at higher levels than seen in differentiated HFKs (Figure 5B). To investigate the role of PPARγ in HPV viral replication and genome maintenance we utilized the drug HX531, which inhibits PPARγ expression [26]. For this analysis, HPV31-positive cells were differentiated in high-calcium media in the presence or absence of HX531 and total DNAs were isolated at 48 hours and 96 hours for Southern blot analysis. As seen in Figure 5C, inhibition of PPARγ by HX531 blocks HPV31 genome amplification. In addition, HX531 treatment did not interfere with stable maintenance of HPV episomes in undifferentiated cells, as shown in Figure 5D. The inhibition may have a modest effect on the expression of involucrin upon differentiation (Figure 5F). To confirm that STAT-5 was indeed a regulator of PPARγ, we examined levels in cells infected by shRNA lentiviruses specifically targeting STAT-5. Inhibition of STAT-5α blocks the expression of PPARγ only upon keratinocyte differentiation while STAT-5β inhibition suppresses the expression of PPARγ in both monolayer and differentiated keratinocytes (Figure 5E). This indicates STAT-5 is an upstream regulator of PPARγ expression.

To investigate whether inhibition of PPARγ by HX531 interferes with DNA damage signaling, HPV31-positive CIN 612 cells were treated with HX531, differentiated in high calcium and whole cell lysates isolated at 48 hours and 96 hours for Western blot analysis. Our studies indicate that treatment with HX531 inhibits PPARγ expression and correspondingly blocks the activation of ATM and CHK2 phosphorylation (Figure 5F). At 48 hours of differentiation, the levels of phosphorylated ATM or phosphorylated CHK2 in HX531-treated cells were comparable to those seen in control differentiating cells. In contrast, after 96 hours of HX531 treatment and differentiation, the levels of phosphorylated ATM and phosphorylated CHK2 were greatly reduced. Interestingly, total ATM levels were also reduced while CHK2 levels decreased modestly. We conclude that suppression of PPARγ by HX531 negatively interferes with DNA damage signaling in HPV positive keratinocytes.

### High-risk oncoprotein E7 is responsible for constitutive STAT-5 activation

**Figure 6:**
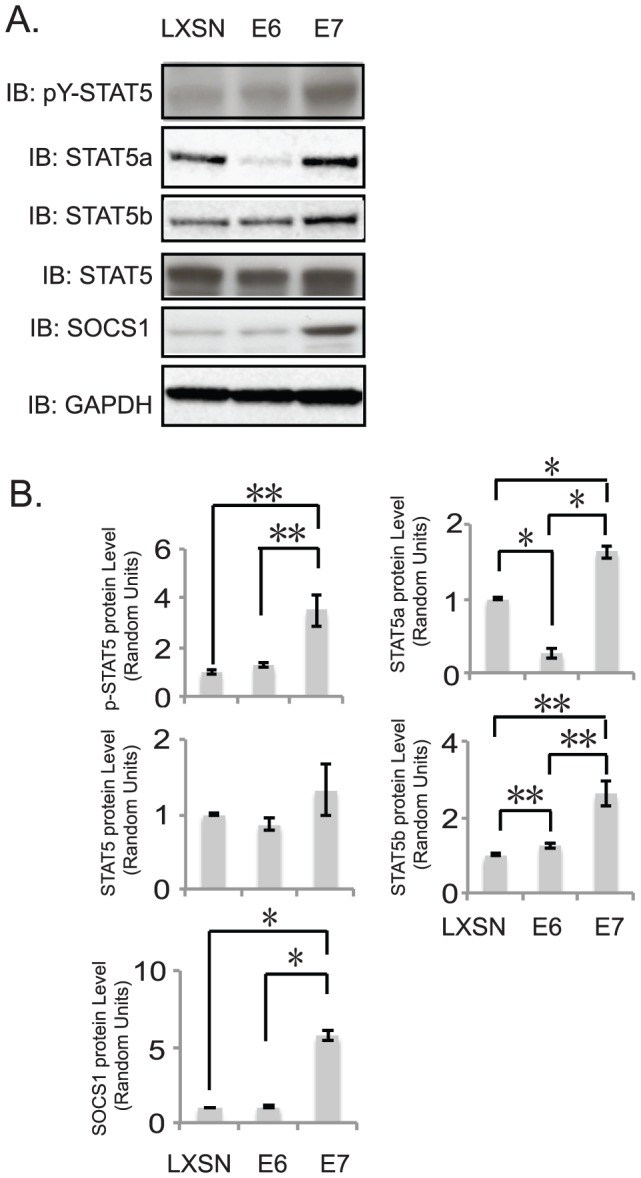
HPV E7 protein is responsible for STAT-5 activation. A) Western blot analysis of p-STAT-5, STAT-5α, STAT-5β, STAT-5, SOCS-1 and CDC25A protein levels in HFK, 31E6, and 31E7cells. B) Bar graphs show the relative expression level of target proteins, normalized to GAPDH, from panel A western analysis. The statistical analysis was assayed by 2-tail t-test. Data = mean +/−standard error. * indicates p-value<0.01; ** indicates p-value<0.05. All results are representative of observations from 3 independent experiments.

HPV oncoprotein E6 and E7 play important roles in cell transformation and immortalization. To determine whether E6 or E7 regulates the activation of STAT-5, we assayed the levels of STAT-5α, STAT-5β, STAT-5, and phospho-STAT-5 by Western blot analysis using primary HFKs persistently infected with retroviruses expressing HPV31 E6 or E7. There were no significant differences in the levels of the total STAT-5 between HFK and E7 cells, however E6 acted to decrease levels of STAT-5. Importantly, the levels of phosphorylated STAT-5 were much higher in E7 expressing cells as compared to HFKs or E6 expressing cells (Figure 6). The suppressor of cytokine signaling 1 (SOCS1) is a known downstream target of STAT-5 [27] and we found it to also be increased in E7-expressing cells. We conclude that E7 is responsible for enhanced phosphorylation of STAT-5 and this is consistent with E7's role in the differentiation-dependent phase of the viral life cycle [2], [3].

## Discussion

Human papillomaviruses must modulate the innate immune response to allow for the establishment of persistent viral infections. Our studies demonstrate that human papillomaviruses activate the innate immune regulator, STAT-5, and that this is necessary for genome amplification in differentiating cells through induction of the ATM DNA damage pathway. Previous studies indicated that HPV proteins suppress expression of STAT-1 to allow for stable maintenance of episomes in persistently infected cells and similar effects were expected for other members of the JAK/STAT pathway. It was therefore surprising that HPV proteins must instead activate STAT-5 to induce productive replication in differentiated cells. STAT-1 and STAT-5 share significant sequence homology, however, STAT-1 is primarily activated by interferons while a broader range of signals including cytokines, growth factors as well as interferons activate STAT-5. Since HPV proteins differentially regulate these factors, the genes targeted by STAT-1 and STAT-5 that are necessary for HPV replication must be distinct. Importantly, STAT-5 activation in cells stably maintaining HPV genomes occurs in the absence of added growth factors or cytokines. This identifies STAT-5 as an important regulator of the HPV life cycle.

While HPV proteins activate STAT-5 in both undifferentiated and differentiated cells, STAT-5 activity is important only for genome amplification and late gene expression in suprabasal cells. It is possible that STAT-5 activation in undifferentiated cells is essential for other activities that are not readily measured in our tissue culture assays. The beta isoform of STAT-5 was shown to be primarily responsible for the effects of STAT-5 on the HPV life cycle and while STAT-5α and STAT-5β share extensive homology, they target different sets of genes. Interestingly, elevated levels of STAT-5β but not STAT-5α have been reported in HPV positive cervical biopsies [28], though the increase we observed in our cell lines is modest.

The major targets of STAT-5 in HPV positive cells were identified as members of the ATM DNA damage pathway linking the JAK/STAT pathway with DNA damage. Activation of the ATM and CHK2 kinases by HPV proteins has been shown previously to be necessary for genome amplification in differentiating cells and we observe that either inhibiting STAT-5 phosphorylation with pimozide or knocking down STAT-5 isoforms with shRNAs blocks activation of this DNA damage pathway. Our studies suggest that CHK2 is the critical factor in DNA damage induced regulation of HPV genome amplification. In our knockdown studies, amplification was found to be dependent upon STAT-5β but not STAT-5α. While p-ATM levels were reduced in the STAT-5α knockdowns, sufficient levels of this kinase were retained or the activity of another kinase such as ATR resulted in high levels of p-CHK2. In contrast, in the STAT-5β knockdowns, no phosphorylation of CHK2 was seen in differentiating cells and this correlated with inhibition of genome amplification. Previous studies using CHK2 inhibitors showed this kinase was important for genome amplification and our studies further suggest it to be a critical regulator. Interestingly, when STAT-5 α and β levels were reduced with shRNAs we also observed a reduction in the levels of total ATM and CHK2 suggesting that STAT-5 can either directly regulate the total levels of these factors in HPV positive cells or stabilize their rate of turnover. STAT-5 does not regulate all members of the ATM pathway as knockdown had no effect on BRCA2 or SMC1. STAT-5 knockdown also reduced the levels of RAD51, consistent with previous studies [29]. In our studies, we observed a modest increase in involucrin levels in STAT-5α knockdowns but no change with STAT-5β knockdowns. This slight increase in involucrin levels may explain the change in genome copy number in monolayer cultures. Importantly, we saw no change in involucrin expression in pimozide treated cells. While we cannot exclude the possibility that STAT-5 could directly activate the HPV late promoter, we have not detected binding to HPV promoter sequences (Gunasekharan and Laimins, unpublished). In addition, STAT-5 does not affect the levels of E6, E7 of E1 early transcripts in cells with episomes.

STAT-5 is a transcription factor that positively and negatively regulates expression of a set of downstream genes. The mechanism by which this transcription factor activates the ATM kinase pathway is therefore likely to be indirect. One straightforward mechanism by which STAT-5 could act would be to regulate expression of a kinase such as JAK2 or alternatively some upstream regulator of ATM activity. One such factor could be TIP60, which is an acetyltransferase that regulates ATM activity [30] and preliminary studies indicate TIP60 levels are decreased in HPV positive cells following pimozide treatment (Hong et al., unpublished). Another factor that is regulated by STAT-5 is the peroxisome receptor, PPARγ and our studies implicate it as an intermediary in activating the ATM DNA damage response. The levels of PPARγ are significantly increased in HPV positive cells and inhibition of PPARγ by the inhibitor HX531 blocks HPV31 genome amplification in a manner similar to that seen with STAT-5 inhibition. In addition, our analysis demonstrated that STAT-5β knockdown inhibited PPARγ expression and correspondingly reduced ATM and pCHK2 (Figure 5F). A role for PPARγ has been previously reported in the regulation of p63 expression [31] and suggested to be a regulator of the DNA damage response [32]. This is in line with our previous findings that p63 activates CHK2 phosphorylation in differentiating HPV-positive cells, which is necessary to induce late viral functions [33].

The E7 protein is be primarily responsible for enhanced phosphorylation of STAT-5. The kinase, AKT, is activated by E7 and previous studies have linked AKT with STAT-5 activation [34], [35]. In preliminary studies, we have blocked AKT activity using inhibitors and found reduced activation of STAT-5 in HPV positive cells along with inhibition of genome amplification (Hong and Laimins, unpublished) suggesting that E7 may act through AKT to activate STAT-5. Interestingly, when E6 is expressed by itself, it induces decreased levels of STAT-5α proteins. When both E6 and E7 are expressed together, the E7 effect is dominant. Similar differential effects of E6 and E7 are seen with other shared cellular targets such as p53 where E6 is the dominant regulator. E7 is not the only HPV protein that can activate the DNA damage response as this activity is shared with the E1 replication protein. When E1 is overexpressed using heterologous promoters it activates a DNA damage response presumably through induction of stalled replication forks [36]–[38]. It is possible that E1 or E5 could also contribute to STAT-5 activation. We previously reported that E7 binds to the active phosphorylated form of ATM but not the unphosphorylated form. This suggests that E7 may direct ATM kinase activity to novel cellular or viral targets in HPV positive cells. The ability of E7 to bind to p-ATM as well as to activate STAT-5 likely provides complementing activities necessary for induction of p-CHK2 and regulation of genome amplification.

Kaposi sarcoma herpes viruses (KSHV) [39] as well as human T cell lymphotropic virus-I (HTLV-I) activate STAT-5 during viral infections [40], while human immunodeficiency viruses (HIV) [41] suppress STAT-5 activity. Interestingly, these viruses also modulate the ATM DNA damage response but a linkage between the two pathways in these viral systems has not been described. An association between STAT-5α and DNA damage has been previously reported by Mallette et al. who used a constitutively activate mutant form of STAT-5α (caSTAT-5) to show that caSTAT-5 induced DNA Damage foci along with enhanced levels of p53 and senescence [42]. In our studies, we did not observe either the induction of p53 or cellular senescence suggesting that ca-STAT-5 and HPV mediated STAT-5 activation function through related but different mechanisms. Overall, our studies indicate that HPV genome amplification is dependent upon the activation of the JAK/STAT transcriptional regulator, STAT-5 and that this is necessary to induce the DNA damage response. This work demonstrates that HPV proteins differentially activate and suppress members of the JAK/STAT pathway to allow for differentiation-dependent productive replication by modulation of the ATM DNA damage pathway.

## Materials and Methods

### Cell culture

Human foreskin keratinocytes (HFKs) were isolated from neonatal foreskins as previously described [43]. Human keratinocytes maintaining HPV31 episomes were generated by transfection of HFKs with viral genomes as previously described [43]. Cells expressing E6 or E7 were generated by infection of HFKs with recombinant retroviruses, as previously described [9]. All HFKs and HPV-positive cells were maintained in E-medium supplemented with mouse epidermal growth factor (5 ng/ml; Collaborative Biomedical Products, Bedford, MA) in the presence of mitomycin C-treated NIH 3T3 J2 fibroblast feeders [43]. To induce differentiation, cells were cultured in keratinocyte basal medium (KBM) with growth supplements for at least 12 h and then switched to KBM (without supplements) containing 1.5 mM CaCl_2_ for up to 96 hours.

### Antibodies

The antibodies used in this study are as follows: anti- STAT-5α and STAT-5β (Sigma, St. Louis, MO); anti-Bcl-XL, anti-Involucrin, anti-GAPDH, and anti-PARP are from Santa Cruz, Santa Cruz, CA); anti-STAT-5, anti-p-STAT-5, anti-CHK2, anti-ATM, anti-p-CHK2 (Thr68), anti-p-ATM (Ser1981), anti-SMC-1, anti-p-SMC-1, anti-BRCA1, anti-p-BRCA1, anti-BRCA2, anti-PPARγ (Cell Signaling, Danvers, MA); anti-RAD51 (Millipore, Hayward, CA); and anti-keratin-10 (Abcam, Cambridge, MA).

### Western blot analysis

HFKs or HPV-positive cells were first rinsed with PBS and then incubated in Versene (PBS containing 0.5 mM EDTA) for 2 minutes to remove J2 feeders at room temperature. The cell lysates were then prepared, separated on SDS-PAGE gels and proteins transferred to membranes as previously described [44]. The membranes were developed using ECL plus or ECL reagents (Amersham, Pittsburgh, PA). Chemiluminescence signals were detected using Eastman Kodak x-ray film. The intensity of the protein bands was quantitated using ImageJ64 software.

### Southern/Northern blot analysis

HPV-positive cells were first incubated in Versene to remove J2 feeders. To isolate total DNA for Southern analysis, cells were lysed in Southern lysis buffer (400 mM NaCl, 10 mM Tris-HCl, 10 mM EDTA), and then incubated at room temperature with 50 µg/ml RNase A (Sigma-Aldrich, St. Louis, MO) and 50 µg/ml proteinase K (Sigma-Aldrich, St. Louis, MO) at 37°C to remove residual RNA and proteins. Total DNA was then isolated by phenol-chloroform extraction and samples were then digested with Xho I. The DNA samples were electrophoresed in a 0.8% agarose gel at 40 V overnight, and then transferred to Gene Screen nylon membranes (Bio-Rad, Hercules, CA) using vacuum transfer according to the manufacturer's protocols. The membrane was then hybridized with radioactive probes, washed, and signal visualized by autoradiography. The intensity of the gel bands was quantitated using ImageJ64 software. For Northern blot analysis, the cells were lysed in STAT-60 (Tel-Test, Friendswood, TX) according to the manufacturer's protocol. The RNA samples were then processed as previously described [45].

### Lentiviral virion production and transduction

MISSION short hairpin RNA (shRNA) lentiviral vectors (Sigma-Aldrich, St. Louis, MO) were used to achieve STAT-5 knockdown in CIN 612 cells. Five STAT-5α or STAT-5β-specific shRNA constructs in a TRC1 or TRC2 plasmid backbones (named constructs sh5α01-05 and sh5β01-05), a “nontarget” construct (scramble), and an HIV-green fluorescent protein (GFP) construct were individually transfected into 293T cells to produce lentiviral particles. 293T cells were grown to 50–60% confluence and transfected with 5 µg shRNA plasmid DNA, 1.66 µg vesicular stomatitis virus G plasmid DNA, and 3.37 µg Gag-Pol-Tet-Rev plasmid DNA using polyethyleneimine (Polysciences, Warrington, PA). Culture medium was changed after 24 hours and the cells were grown for an additional 48 hours (3 days total). Supernatants were collected and filter sterilized using 0.45 µm syringe filters (Pall, Ann Arbor, MI). CIN 612 cells were incubated with 5 ml viral supernatant consisting of STAT-5 shRNA or scram shRNA control lentiviral particles in the presence of 4 µg/ml hexadimethrine bromide (Polybrene; Sigma-Aldrich, St. Louis, MO) for 72 hours at 37°C. At 24 hours posttransduction, fresh E medium was added to each plate of transduced CIN 612 cells and STAT-5 knockdown was confirmed by Western blot analysis.

## Supporting Information

Figure S1 Knockdown of STAT-5 suppresses ATM DNA damage responses. The figures show the quantification of the band intensities of the western blot analysis shown in Figure 4 and presented as bar graphs. The band intensities were determined by ImageJ64 software. The graphs show the relative expression level of target proteins normalized to GAPDH. A) Quantification of western blot analysis of p-ATM, ATM, p-CHK2, CHK2, and involucrin relative protein levels in uninfected and shRNA lentivirus infected CIN612 cells upon differentiation in high-calcium media for indicated times. B) Quantification of western blot analysis of p-CHK2, CHK2, STAT-5α, and STAT-5β relative protein levels in shRNA control and shRNA lentivirus infected CIN612 cells upon differentiation in high-calcium media for indicated times. C) Quantification of western blot analysis of BRCA-1. p-BRCA-1, and RAD51 relative protein levels in uninfected and shRNA lentivirus infected CIN612 cells upon differentiation for indicated times. The band intensities were determined by ImageJ64 software. The graphs show the relative expression level of target proteins normalized to GAPDH. The statistical analysis was assayed by 2-tail t-test. Data = mean+/−standard error. * indicates p-value<0.01; ** indicates p-value<0.05. All results are representative of observations from 3 independent experiments.
(EPS)

Figure S2 STAT-5-dependent ATM DNA damage signaling may be mediated by PPARγ. The figures show the quantification of the relative band intensities from Figure 5F and plotted in corresponding graphs. The bar graphs show the relative expression level of target proteins, normalized to GAPDH and determined by ImageJ64 software. The statistical analysis was assayed by 2-tail t-test. Data = mean+/−standard error. * indicates p-value<0.01; ** indicates p-value<0.05. All results are representative of observations from 3 independent experiments.
(EPS)
